# Oscillator Selection Strategies to Optimize a Physically Unclonable Function for IoT Systems Security

**DOI:** 10.3390/s23094410

**Published:** 2023-04-30

**Authors:** Raúl Aparicio-Téllez, Miguel Garcia-Bosque, Guillermo Díez-Señorans, Santiago Celma

**Affiliations:** Group of Electronic Design (GDE), Aragón Institute of Engineering Research (I3A), University of Zaragoza, 50009 Zaragoza, Spain

**Keywords:** authentication, cybersecurity, field programmable gate array, identification, Internet of Things, physically unclonable function, ring oscillator, wireless sensor networks

## Abstract

Physically unclonable functions avoid storing secret information in non-volatile memories and only generate a key when it is necessary for an application, rising as a promising solution for the authentication of resource-constrained IoT devices. However, the need to minimize the predictability of physically unclonable functions is evident. The main purpose of this work is to determine the optimal way to construct a physically unclonable function. To do this, a ring oscillator physically unclonable function based on comparing oscillators in pairs has been implemented in an FPGA. This analysis shows that the frequencies of the oscillators greatly vary depending on their position in the FPGA, especially between oscillators implemented in different types of slices. Furthermore, the influence of the chosen locations of the ring oscillators on the quality of the physically unclonable function has been analyzed and we propose five strategies to select the locations of the oscillators. Among the strategies proposed, two of them stand out for their high uniqueness, reproducibility, and identifiability, so they can be used for authentication purposes. Finally, we have analyzed the reproducibility for the best strategy facing voltage and temperature variations, showing that it remains stable in the studied range.

## 1. Introduction

In recent years, the development of the Internet of Things (IoT) has given rise to the exchange of a large amount of data among devices. Wireless Sensor Networks (WSNs) collect sensitive information through sensors strategically distributed in a certain environment. Physically Unclonable Functions (PUFs) can be used to authenticate nodes of WSNs so that only authorized nodes have access to the network and therefore, to the information. The evident need to protect these data against cyberattacks has made PUFs emerge as a promising solution for IoT device authentication, since they do not use any cryptographic resources on the device [1,2].

PUFs generate a response that depends on stochastic variations that occur during the manufacturing process of the devices, which makes them ideal for identification, authentication, and key generation purposes. They also eliminate the need to store secret information in non-volatile memories, as they only generate a key when it is necessary for an application, increasing the security of the IoT systems [3,4,5]. Since the term PUF was suggested in 2001 [6], more than forty types of PUFs have been proposed, including Arbiter PUFs [7] and metastability PUFs [8,9]. This work focuses on Ring-Oscillator PUFs (RO-PUFs) implemented in FPGAs [10,11], based on the proposal of Suh and Devadas in 2007 [12]. Although another type of PUF could be studied, RO-PUFs are selected for their suitability to be implemented in FPGAs, and while in an ASIC it is possible to exploit the design layout techniques, in an FPGA these characteristics are limited to the available resources, such as Look-Up Tables (LUTs), Block RAMs, or Flip-Flops. However, RO-PUFs are easy and low-priced to implement in FPGAs as they only use logic gates.

In a RO-PUF, the response is obtained by comparing the frequencies of identical ring oscillators. Consequently, some architectures of RO-PUFs require to implement ring oscillators in different locations of the FPGA. Indeed, it has already been observed that the frequencies of the oscillators have a certain correlation with the location in which they are implemented in the FPGA [13,14]. If the locations of the oscillators are not selected carefully, some comparisons may lead to predictable rather than random bits. The effect of location has already been analyzed in other kinds of PUFs such as the DD-PUF [15] or the XOR-PUF [16].

In this work, we propose an approach to minimize edge effects and other effects related to physical separations existing in the FPGA, based on fixing the routing of the oscillators. However, fixing the routing does not eliminate other effects such as the difference between Slices(0) and Slices(1) in the FPGA, or the existence of a negative spatial correlation. This means that some oscillators may have systematically higher frequencies than others. As a result, the bits that result from the comparison of these oscillators will be predictable rather than random. Therefore, it is necessary to carefully select the locations of the oscillators to be compared. For this purpose, we propose five strategies to select the ring oscillators to be compared, including a novel strategy which avoids the need to fix the routing of the oscillators, since this task is performed manually by the designer and can be time-consuming. In addition, the best strategy is analyzed in terms of reproducibility, uniqueness, and identifiability; as well as the stability of the PUF facing environmental changes, such as voltage or temperature variations.

This paper is divided in six sections. Section 2 shows the architecture of the RO-PUF implemented and some metrics to determine its quality. In Section 3, we explain the methodology used and we compare the frequencies of the oscillators with fixed routing and with automatic routing. Furthermore, some strategies to select the oscillators to compare are explained. In Section 4, the responses of the PUF with the proposed strategies are shown and discussed in terms of reproducibility, uniqueness, and identifiability. Section 5 analyzes the effect of variations in the temperature and the supply voltage of the FPGA in a PUF based on the best strategy. Finally, in Section 6, the conclusions are shown.

## 2. Background

### 2.1. Architecture of a RO-PUF

**Figure 1:**
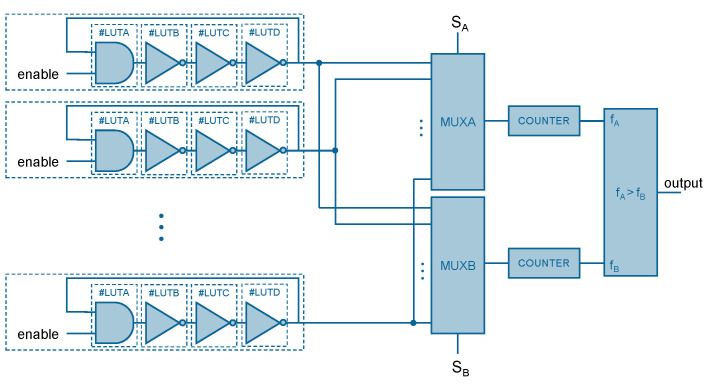
Schematic of the RO-PUF architecture implemented in this work.

A Ring Oscillator (RO) is formed by an odd number of inverters in a ring. Furthermore, an AND gate is usually added as an enable. The architecture of RO-PUF used in this work is based on the one proposed and analyzed in [17]. This architecture is formed mainly by identical ring oscillators and frequency counters (Figure 1). Furthermore, to achieve a more stable response, the outputs of the oscillators are connected to two multiplexers (MUX). The frequencies are compared in pairs, so that if the frequency of the first RO is larger than the frequency of the second RO, the output bit is 1; and 0 otherwise [3,12,18].

If all comparisons are performed, a set of comparisons which is not linearly independent is obtained. To exemplify this, imagine the comparison of three oscillators 1, 2, 3 with frequencies $f_{1}<f_{2}$ and $f_{2}<f_{3}$. Consequently, $f_{1}<f_{3}$ and the comparison between RO-1 and RO-3 will be predictable. Typically, not all comparisons are used to implement a RO-PUF. In fact, there are several RO-PUF architectures depending on the comparison pattern used. In this work, the 2-masking architecture has been used, which consists of comparing the oscillators in pairs so that any oscillator is repeated in each comparison [19].

### 2.2. PUF Key Metrics

Two properties can be analyzed to determine the quality of a RO-PUF: reproducibility and uniqueness [4]. To measure these properties, we have used the Hamming Distance as figure of merit. Given two binary words *Y* and $Y^{\prime }$ of the same length *n*, the Hamming Distance (*HD*) is defined as the XOR operation between the bits $Y_{i}$, $Y_{i}^{\prime }$ of both sequences:

(1) $$HD\;\left(Y;Y^{\prime }\right)=\sum_{i=1}^{n}Y_{i}\;\mathrm{XOR}\;Y_{i}^{\prime }.$$

- Reproducibility: analyzes whether a PUF always provides the same response for the same FPGA. It is measured with the intra-*HD*. A PUF with perfect reproducibility will present an average intra-*HD* of 0%.
- Uniqueness: analyzes whether the same PUF provides different responses for each FPGA. It is measured with the inter-*HD*. A PUF with perfect uniqueness will present an average inter-*HD* of 50%.

This last point exemplifies the importance of using oscillators with similar frequencies. In fact, one of the challenges that a designer who pretends to implement a RO-PUF must face is to select the locations of the oscillators. The information existing in the literature in this regard is scarce or even contradictory. On the one hand, some authors propose to place the oscillators as close as possible to each other, and make the comparisons between adjacent oscillators to avoid creating a delay pattern in the components of the matrix. This effect is caused by intra-die variations [20]. Other authors show that implementing oscillators in adjacent locations increases the probability of ring oscillators coupling with each other, making the PUF useless. This justifies the importance of avoiding, as far as possible, using oscillators implemented close in the FPGA [21].

Furthermore, to determine whether the proposed strategy is suitable for authentication purposes, the identifiability of the PUF has been studied. It is evident that, in those systems, false acceptances and false rejections must be avoided. The probability of an identification attempt to result in a false rejection is measured by the false rejection rate:

(2) $$FRR=F_{\mathrm{binom}}\left(p^{\mathrm{intra}}\right)$$

i.e., the cumulative distribution function (CDF) of the intra-*HD* distribution $p^{\mathrm{intra}}$. The probability of an identification attempt to result in a false acceptance is measured by the false acceptance rate:

(3) $$FAR=1-F_{\mathrm{binom}}\left(p^{\mathrm{inter}}\right)$$

i.e., the CDF of the inter-*HD* distribution $p^{\mathrm{inter}}$. Regarding the identifiability of the PUF, *FAR* and *FRR* should be as small as possible, but they cannot be minimized at the same time. Thus, it is necessary to reach a trade-off between the PUF being robust and safe at the same time. For this purpose, we use the equal error threshold $t_{EER}$, defined as the discrete threshold for which the inter-*HD* and the intra-*HD* distributions intersect; as well as the equal error rate *EER*, defined in Equation (4) [4]. The *EER* is the most important property of the PUF, as it provides an objective way to determine whether the PUF is suitable for authentication purposes. Thus, it will be used as figure of merit. The lower the *EER*, the better the PUF will be in terms of identifiability.

(4) $$EER=max\{FAR\left(t_{EER}\right),FRR\left(t_{EER}\right)\}$$

In this work, we have analyzed the frequencies of several oscillators to determine which is the best strategy to select the oscillators. Five strategies have been proposed and compared. Using the best strategy, we have obtained a RO-PUF with an intra-*HD* lower than 2%, an inter-*HD* close to 48%, and an equal error rate of $10^{-11}$ orders of magnitude.

## 3. Methods

### 3.1. Description of the Experimental Setup

**Figure 2:**
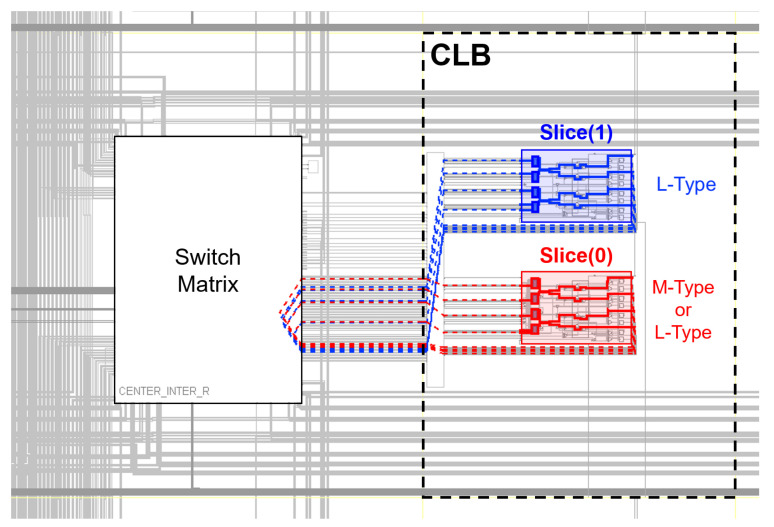
Architecture of a CLB. Each CLB contains two types of slice: Slice(0) and Slice(1). Slice(0) corresponds to slices with even x-coordinates while Slice(1) corresponds to slices with odd x-coordinates. The routing of the ROs is different depending on the type of slice used.

This experiment has been performed using PYNQ-Z2 boards, which include Zynq 7000 ARM/Artix 7 FPGAs [22]. The FPGA is provided with 13,300 slices arranged in pairs. Each slice contains four 6-input LUTs: A, B, C, and D; and 8 flip-flops. Furthermore, as it can be seen in Figure 2, each Configurable Logic Block (CLB) contains a pair of slices: Slice(0), situated in the bottom part of the CLB; and Slice(1), situated in the upper part. The slices are expressed with different coordinates $X_{i}Y_{j}$.

**Figure 3:**
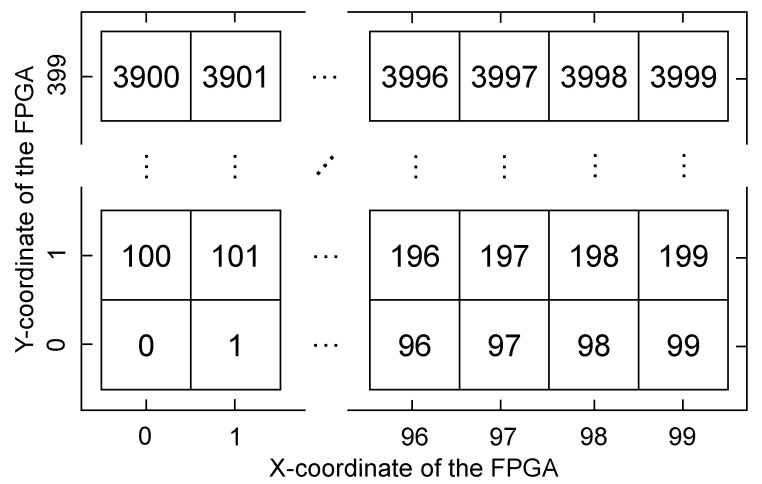
Definition of the indices of the oscillators and their approximate location in the FPGA.

In this analysis, 4000 3-LUT identical ring oscillators have been implemented in 40 FPGAs. Each oscillator has been implemented in one slice using 3 LUTs: B, C, and D. In addition, the oscillators have been arranged in a 40 × 100 matrix and an index has been assigned to each RO, starting with RO-0 in the slice $X_{0}Y_{0}$ to RO-3999 in the slice $X_{99}Y_{399}$. Figure 3 shows the assigned index of the oscillators and their approximate location in the FPGA. Furthermore, an AND gate has been implemented in LUT-A of every slice as an enable, so that in every frequency measurement only the output of the oscillator to be measured will be oscillating, while in the rest of oscillators, it will be stable. This avoids the possible effect of frequency coupling described in [21].

### 3.2. Routing of the Oscillators

**Figure 4:**
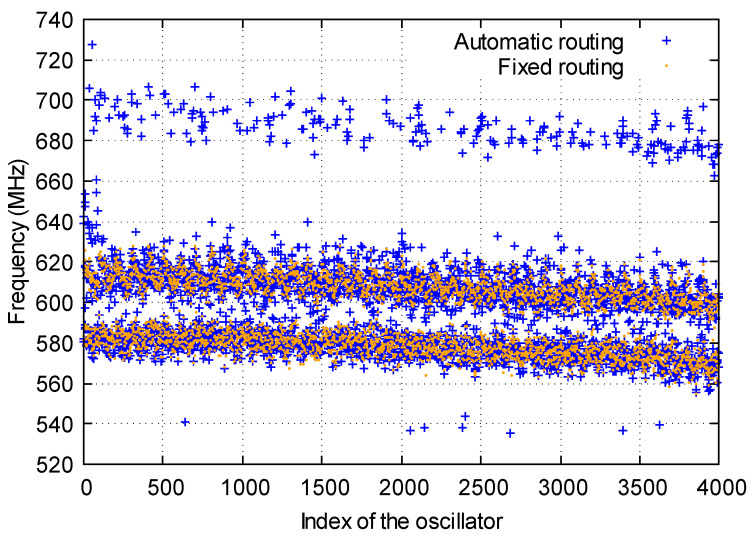
Frequency of the oscillators and their assigned index for oscillators.

One of the goals of this work is to analyze the frequencies of the oscillators depending on some parameters such as the location, the routing of the oscillators, or the type of slice where they are implemented to determine the optimal way to construct a RO-PUF in an FPGA. For this purpose, the frequency of the same RO structure over 4000 different locations has been measured. In Figure 4, we have represented in blue the frequencies of the oscillators against their assigned index.

As it can be seen, there is a certain variability in the frequency of the oscillators depending on their position in the FPGA:

(i) Two frequency domains: oscillators implemented in Slices(1) have higher frequencies than those implemented in Slices(0). These two types of slices use different routing resources, causing the oscillators to have different frequencies. PUFs in the literature base their results on comparisons of identical oscillators. This effect may cause a novel designer to compare close oscillators to design a PUF, without realizing that they are systematically comparing fast oscillators with slow oscillators.
(ii) Negative correlation: there is a negative correlation between the frequency of the oscillator and the assigned index. As the index of the oscillator increases, the frequency tends to decrease. This means that oscillators close to the $X_{0}Y_{0}$ coordinate have higher frequencies compared to those close to the $X_{99}Y_{39}$ coordinate [13]. This could be a consequence of the intra-die variations occurring during the manufacturing process of the devices. Furthermore, this could also be a consequence of the power lines of the FPGA, which cause some LUTs to arrive at a different power supply voltage, altering the oscillator frequencies. For this purpose, we have measured the frequencies of the oscillators for various voltages and we represented the frequencies against the assigned indices. We have seen that the negative correlation tendency remains unalterable for all voltages, ruling out this effect.
(iii) High- and low-frequency ROs: some oscillators tend to have larger frequencies (near 700 Hz). Furthermore, there are a few oscillators which tend to have lower frequencies (near 540 Hz). Significant differences have been observed in the routing of these oscillators which could explain this phenomenon.
(iv) Edge effects and physical separations: oscillators implemented in the edge of the FPGA tend to have higher frequencies compared to other oscillators. Furthermore, oscillators located near physical separations of slices in the FPGA tend to have a different routing compared to the oscillators implemented in other locations of the FPGA, leading to different frequencies.

Some authors have already pointed to the importance of carrying out a uniform routing to obtain a good PUF in terms of uniqueness [23]. To minimize all the effects related to automatic routing carried out by Vivado, the routing of the oscillators has been fixed before the implementation phase. In this work, all oscillators implemented in Slices(0) will use analogous routing resources, and either all oscillators implemented in Slices(1) [24]. An example of the routing resources used for each type of slice is shown in Figure 2.

Subsequently, we have measured the frequency of the oscillators with their routing fixed and we have represented the results in orange together in Figure 4. As it can be seen, by fixing the frequency of the oscillators, some effects caused by the edge of the FPGA or the physical separations disappear. However, we still observe two clear frequency domains corresponding to oscillators implemented in Slices(0) and Slices(1), as well as a negative correlation between the frequency of the oscillator and the assigned index. Furthermore, an additional effect is also observed between ROs implemented in M-type and L-type LUTs. This will by analyzed in the following section.

### 3.3. Differences between LUTs M AND L

**Figure 5:**
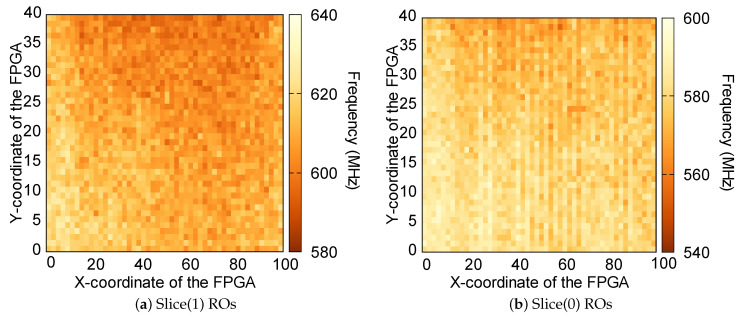
Frequency of the oscillators depending on their location in the FPGA for oscillators in (**a**) Slices(1) and (**b**) Slices(0).

In this section, we take a closer look at other possible effects beyond the routing of the oscillators implemented in Slices(0) and Slices(1) separately. For this purpose, we have represented the frequencies of oscillators implemented in both types of slices depending on the coordinate of the FPGA where they have been implemented. As it is shown in Figure 5, there is a certain spatial correlation in both the x-axis and y-axis. Since the types of slices vary depending on the x coordinate, we have decided to study in more detail the correlation in the x-axis.

**Figure 6:**
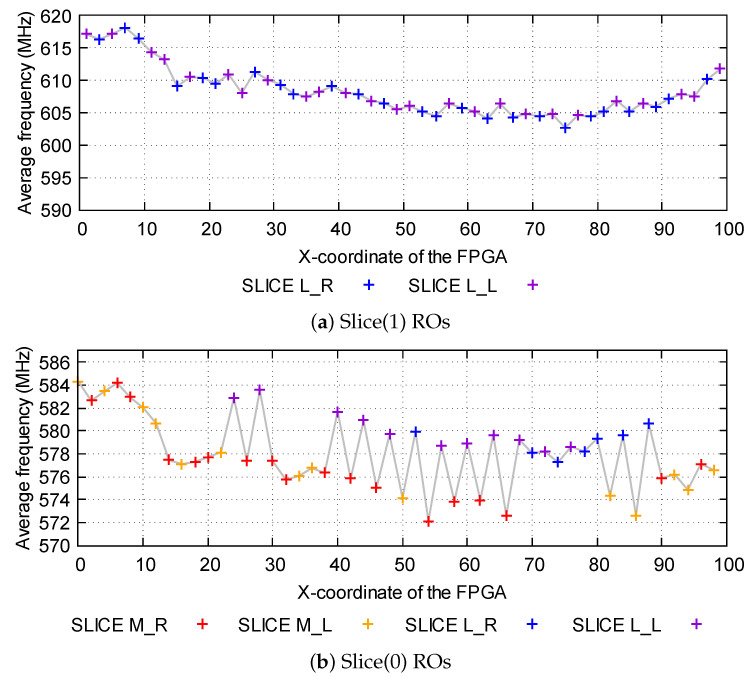
Average frequency of the oscillators depending on the coordinate of the slice where they are implemented for oscillators in (**a**) Slices(1) and (**b**) Slices(0). Furthermore, LUTs can be M-type or L-type and CLBs can be identified as left (L) or right (R).

For this purpose, in Figure 6 we have represented the average frequency of the oscillators implemented in the same x-coordinate of the FPGA, for oscillators in Slices(0) and Slices(1). Two effects have been mainly observed:

- Oscillators implemented from the slice with $X_{0}$ coordinate to the slice with $X_{13}$ coordinate, present higher frequencies compared to the rest of oscillators of the same domain. This can be seen as an edge effect, since all these oscillators correspond to the left part of the board. Some articles in the literature [20] already observe that, in other types of FPGAs, the oscillators located on the left side of the FPGA tend to have different frequencies than other oscillators.
- Oscillators implemented in L-type LUTs tend to have higher frequencies compared to oscillators implemented in M-type LUTs. The main difference between the two types of LUTs is that L-type LUTs can only implement logic functionalities. However, M-type LUTs can also be configured to implement distributed memory or shift registers. Nevertheless, the manufacturer does not provide any type of information to explain this difference.

Furthermore, in the Zynq 7000 SoC, CLBs are named differently depending on whether they are located in the left part of the switchbox (in that case they are named CLB_L) or in the right part (where they are named CLB_R). Subsequently, we have two types of CLBs (L or R), two types of slices (0 or 1), and two types of LUTs (M or L).

In order to analyze whether there is any difference between oscillators implemented in CLBs_L or CLBs_R, the average frequency of all oscillators located in M_L, M_R, L_L, and L_R slices has been obtained. As it can be seen in Table 1, oscillators located in CLBs_L tend to have a slightly higher frequency than the oscillators located in CLBs_R. This difference is larger for oscillators in Slices(1) than those implemented in Slices(0), where the difference is negligible. However, it must be noticed that other effects such as negative correlation are not being taken into account when performing the average frequency.

As these effects are less relevant compared to the difference of frequency between Slices(1) and Slices(0), we have not taken into account the type of LUT and the type of CLB when designing the selection strategies.

### 3.4. Selection Strategies of Oscillators

As it has been observed in the previous section, by fixing the routing of the oscillators we eliminate some effects caused by the architecture of the FPGA. However, some differences are still observed between the frequencies of the oscillators implemented in different types of Slices, LUTs, or CLBs, as well as the existence of a negative spatial correlation in the FPGA. Therefore, it is necessary to define some selection strategies of the locations of the oscillators to be compared to increase the randomness of the PUF response.

In this experiment, we have implemented a RO-PUF formed by 200 identical ring oscillators, using as comparison strategy the 2-masking architecture. In this situation, the designer could choose among 4000 locations. In this paper, five strategies are proposed to select 200 oscillators over a set of 4000 locations:

1. 200 first: this strategy consists of using the oscillators with the first 200 indices: oscillator 0, oscillator 1 to oscillator 199.
2. 200 random: in this strategy, we have selected 200 oscillators randomly. Furthermore, comparisons have been performed randomly as well. Taking into account that the oscillator index is a random variable, statistically, half of the oscillators will be in Slices(0) and the other half will correspond to Slices(1).
3. 200 random same-domain: this strategy consists of selecting 200 oscillators randomly among those in the same frequency domain. Comparisons have been performed in random order too. Using this strategy, the oscillators will have similar frequencies. Therefore, when comparing the oscillators in pairs, the output bit is expected to be less predictable compared to the previous strategies.
4. 200 first same-domain: in this strategy, we have selected the first 200 oscillators implemented in Slices(1): oscillator 1, oscillator 3 to oscillator 399. The idea behind this strategy is to reduce the effect of negative spatial correlation of the frequencies of the oscillators by selecting oscillators situated close in the FPGA.
5. 200 optimal: this strategy consists of selecting oscillators with similar frequencies in order to avoid comparisons of Slices(0) and Slices(1) oscillators. For this purpose, we have calculated the average frequency of the oscillators in the same frequency domain and its error. Subsequently, we have analyzed 20% of the FPGAs to obtain the indices of the oscillators whose frequency is in the interval:
  (5) $$\left(\bar{f}-\alpha \;\sigma_{\bar{f}}\;,\;\bar{f}+\alpha \;\sigma_{\bar{f}}\right)$$
  where $\bar{f}$ is the average frequency of the oscillators in one FPGA and $\sigma_{\bar{f}}$ is the standard error of the mean. In this way, we have used a small sample of the available FPGAs to study whether the locations obtained with this strategy are transferable to other instances. The parameter α is a real number generated by trial and error so that, at the end of the process, there are exactly 200 oscillators. Using this strategy, we are selecting oscillators with similar frequencies, eliminating the problem of selecting adjacent oscillators. In addition, setting the routing of the oscillators can sometimes be complicated, especially for oscillators with a large number of inverters since you have to manually select the routing resources of the oscillators. The very interesting aspect about this strategy is that it can be carried out using an automatic routing, since it selects oscillators with similar frequencies and does not take into account the existence of oscillators with highly different frequencies from the rest. Therefore, the effect of automatic routing and the effect of the difference between L-type and M-type slices are avoided.

The 200 random odd, 200 first odd, and 200 optimal strategies are defined to avoid the comparisons of oscillators implemented in Slices(0) and those implemented in Slices(1), which led to predictable rather than random results, as we have shown.

## 4. Results

In this section, a RO-PUF has been implemented with each strategy. We have analyzed the implemented PUF in terms of uniqueness, reproducibility, and identifiability. Furthermore, the five strategies are compared to determine which is the best strategy to select the oscillators.

### 4.1. Uniqueness

In order to analyze the uniqueness of the RO-PUF, we measured the frequency of the 4000 oscillators in all the five strategies described above, in 40 different FPGAs. Based on these measurements, in post-processing, the 100-bit responses that would be obtained with each strategy have been simulated. In this way, one response is obtained for each FPGA. Subsequently, with those answers, we have calculated the inter-*HD*. The average inter-*HD* and the standard error obtained for each strategy is shown in Table 2.

As it can be seen, all the average inter-*HD* values are below the theoretical 50% in the five strategies analyzed, even considering the standard errors of the mean. Furthermore, the inter-*HD* drastically changes depending on the strategy used:

1. 200 first: with this strategy, a low inter-*HD* has been obtained, since oscillators in the high frequency domain are systematically being compared to oscillators in the low frequency domain. As this phenomenon occurs in all the studied FPGAs, the response is predictable rather than random.
2. 200 random: with this strategy, a low inter-*HD* is still obtained compared to the theoretical inter-*HD*, since about half of the comparisons are between oscillators in the high frequency domain and in the low frequency domain. This causes half of the bits in the output to be predictable.
3. 200 random same-domain: using this strategy, the inter-*HD* rises to 36.08 ± 0.27%. This value is clearly closer to the expected 50%. When selecting oscillators of the same domain, those with closer frequencies are being compared, causing the inter-*HD* to rise by about 17% compared to the previous strategy. However, as we have demonstrated before, when the index of the oscillator increases, the frequency decreases. As a result, comparisons of "far" oscillators yield predictable bits with high probability.
4. 200 first same-domain: using this strategy, the inter-*HD* increases drastically to 47.79%, since we are selecting oscillators with closer frequencies, as all of them belong to the high frequency domain. Furthermore, the effect of the negative correlation explained before is greatly diminished. These two effects cause an increase in the randomness in the response. It must be noted that, using the automatic routing done by Vivado, some oscillators in the high domain frequency had a clearly different frequency compared to the rest of oscillators. In this case, the designer may have to eliminate these oscillators before taking the 200 first same-domain oscillators. However, as in this case, when the routing is fixed this problem disappears, as all oscillators implemented in Slices(0) or Slices(1) belong to a clearly defined domain.
5. 200 optimal: the highest inter-*HD* is obtained with this strategy. Furthermore, it corresponds with the closest value to the theoretical inter-*HD*. Therefore, 200 optimal is the best strategy in terms of uniqueness as it increases the randomness of the response, providing a more secure key. This is because, when selecting the common oscillators of some FPGAs with a frequency closer to the average, oscillators with similar frequencies are being selected. The main drawback of this method is that it requires a prior study of the frequencies of the oscillators based on their position in a small group of FPGAs. In a real scenario, the enrollment phase could be used to carry out this study, as it is shown that it provides an important rise in the uniqueness of the PUF.

As explained before, the theoretical inter-*HD* should be a binomial distribution with parameters n=100 and p=0.5. Table 2 shows the binomial parameter *p* obtained in the five different scenarios. As it can be seen, the parameters obtained are comparable to the average inter-*HD*.

Table 2 shows the $D_{\mathrm{K-S}}$ obtained for each strategy. As it can be seen, 200 first and 200 random present the highest $D_{\mathrm{K-S}}$. In the 200 random same-domain strategy, the $D_{\mathrm{K-S}}$ remains high, near to 80%. For the 200 first same-domain strategy, the $D_{\mathrm{K-S}}$ decreases drastically to 15.12%. However, the lower value is obtained for the 200 optimal strategy as expected. This result represents an improvement of the uniqueness of the PUF of 3% compared to the 200 first same-domain strategy.

**Figure 7:**
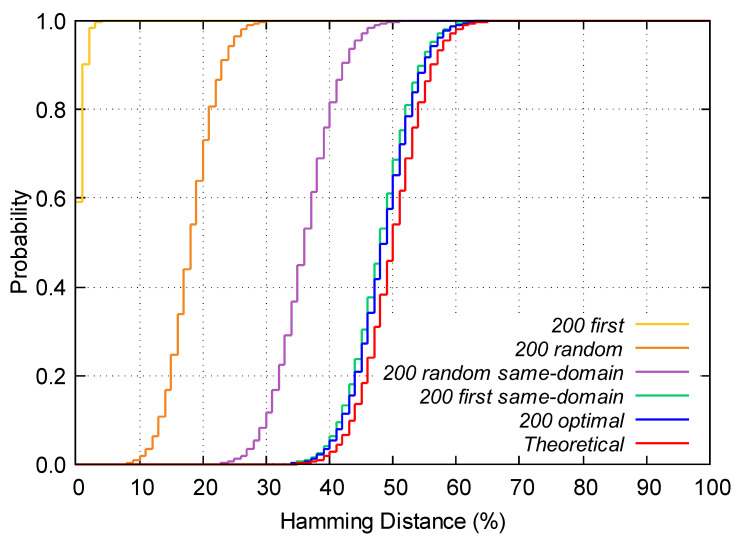
Comparison of the cumulative distribution probabilities (CDF).

To quantify whether the experimental distribution obtained fits to the binomial distribution expected, we have carried out a Kolmogorov–Smirnov test [25]. Figure 7 shows the comparison of the CDFs of the inter-*HD* using the five strategies compared to the CDF of the theoretical distribution.

In this test, we have used an additional parameter to analyze the uniqueness of the PUF using the different strategies: the Kolmogorov–Smirnov statistic $D_{\mathrm{K-S}}$, defined in (6). The best strategy will present an average inter-*HD* closer to 50% and a lower $D_{\mathrm{K-S}}$.

(6) $$D_{\mathrm{K-S}}=\underset{0\leq i\leq 100}{max}\left|{\hat{F}}_{n}\left(x_{i}\right)-F_{0}\left(x\right)\right|$$

From the experimental results obtained in this experiment, it can be assumed that 200 first optimal and 200 first same-domain are the best strategies in terms of uniqueness.

### 4.2. Reproducibility

In this section, the intra-*HD* has been used to determine the reproducibility of the RO-PUF. For this purpose, we have measured the frequency of the 4000 oscillators 100 times in five different FPGAs. As in the previous case, based on these measurements, the response that the implemented PUF would generate has been simulated. Table 3 shows the average intra-*HD* obtained with each strategy and the standard error.

As explained before, perfect uniform random responses should have an average intra-*HD* of 0%. As it is shown, intra-*HD* varies greatly depending of the FPGA.

Firstly, using the 200 first oscillators, an intra-*HD* of 0% is observed, meaning that the RO-PUF has a perfect reproducibility. However, despite the high reproducibility obtained, it has been already shown that this strategy presents a low uniqueness.

Secondly, a higher intra-*HD* is observed in the 200 random and 200 random same-domain strategies compared to the 200 first strategy. This is because, when selecting oscillators with similar frequencies, small variations of temperature and supply voltage cause some bits to change from 0 to 1 and vice versa, increasing the intra-*HD*. However, the average intra-*HD* obtained using both strategies does not exceed 2% in any FPGA, meaning that the reproducibility of the PUF is slightly reduced compared to the 200 first strategy but they still present a high value. Despite the high reproducibility obtained, both strategies present a low uniqueness.

Finally, the highest intra-*HD* is obtained with the 200 first same-domain and 200 optimal strategies. However, the average intra-*HD* obtained using both strategies barely exceeds 2.5% is all FPGAs, meaning that the reproducibility is reduced compared to the previous strategies but they still present a low intra-*HD*. To conclude, it must be noticed that the intra-*HD* obtained with the 200 optimal strategy is slightly higher compared to the 200 first same-domain strategy and therefore presents a worse reproducibility.

### 4.3. Identifiability

Since the strategies with better reproducibility have worse uniqueness and vice versa, we have analyzed the identifiability, which is very useful since it makes it possible to determine which is the best strategy in an objective way. We have calculated the *FAR* and *FRR* for all possible thresholds and then we have chosen the threshold for which the two errors are as similar as possible. Furthermore, we have obtained the equal error rate *EER* and the identification threshold $t_{\mathrm{EER}}$.

As it is shown in Table 4, the highest *EER*s are obtained for the 200 first strategy. Therefore, using this strategy, we obtain a PUF with low identifiability so it could not be used for device identification purposes. Regarding the 200 random strategy, it increases the identifiability of the PUF by five orders of magnitude compared to the previous strategy. However, a high *EER* is still observed. Regarding the 200 random same-domain strategy, the *EER* is reduced by four orders of magnitude with regard to the previous strategy obtaining an *EER*$∼10^{-9}$, so it could be considered for device authentication purposes. Although we could begin to consider this strategy to authenticate the devices of a WSN, there are still two strategies which exhibit a higher identifiability.

Finally, for the 200 first same-domain and 200 optimal strategies, a similar and low *EER* is obtained. In both cases, a high identifiability is observed obtaining an *EER*$∼10^{-11}$ and therefore, they are the best strategies to be used for device authenticating purposes.

**Figure 8:**
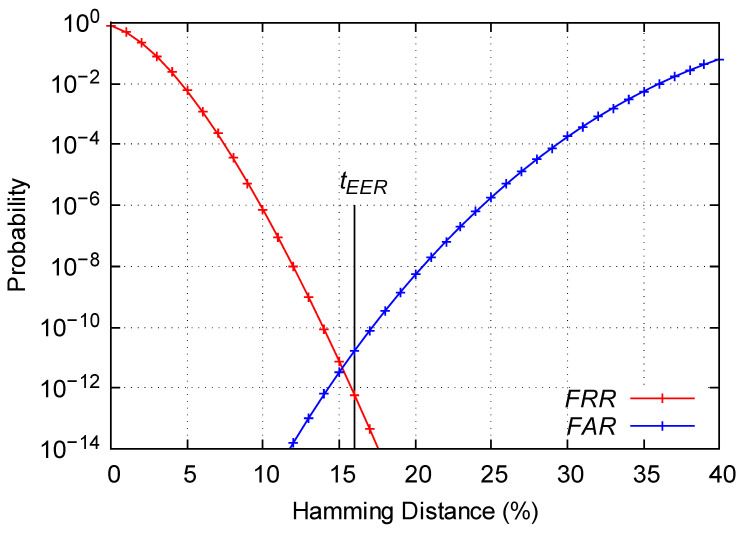
Values of *FAR* and *FRR* for the 200 first same-domain strategy.

**Figure 9:**
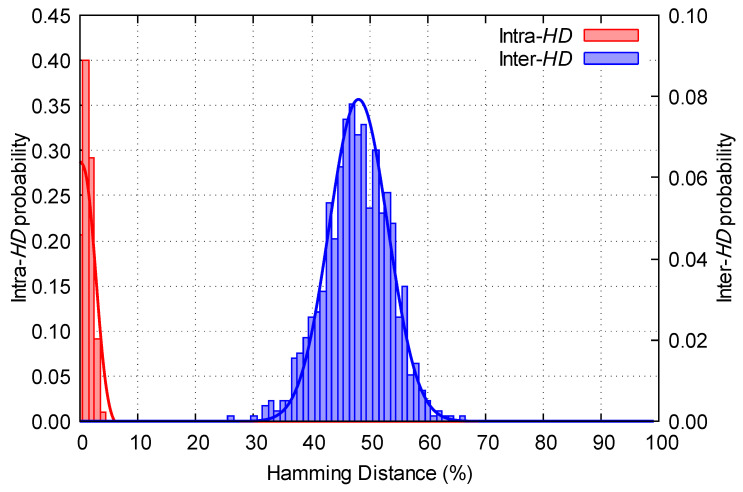
Intra-*HD* and inter-*HD* for the 200 first same-domain strategy.

Therefore, 200 first same-domain and 200 optimal are the best strategies regarding the identifiability of the RO-PUF. Both strategies provide great results in terms of identifiability. However, as the 200 first same-domain strategy does not need a prior analysis of the frequencies of the oscillators, which could be inconvenient for some applications. Consequently, this strategy will be taken in the following section as the best strategy to analyze the stability of the RO-PUF facing environmental changes. Figure 8 shows the *FAR* and *FRR* curves for the 200 first same-domain strategy. Furthermore, in Figure 9 we have represented the intra-*HD* and inter-*HD* together for the 200 first same-domain strategy in one FPGA.

## 5. Voltage–Temperature Variations

Variations in the operating environment may cause the RO-PUF to provide different responses to the same challenge. Therefore, the response of the RO-PUF under temperature and FPGA supply voltage changes has been studied for the 200 first same-domain strategy.

### 5.1. Temperature Variations

To study the effect of temperature in the performance of the RO-PUF, we have used a thermal chamber Aralab FitoTerm 22E, which makes it possible to vary the temperature from −40 to 160 °C. For this purpose, we have measured the response of the RO-PUF at different temperatures with respect to the room temperature (20 °C). Firstly, among the 100 binary sequences obtained at 20 °C, we have obtained the mode of the distribution, i.e., the sequence of bits which is most repeated. This sequence will be our “golden key”. Secondly, the hamming distance between the responses of the PUF at different temperatures and the “golden key” has been calculated. This process has been repeated for each one of the 100 measurements made at each temperature, and the average intra-*HD* has been obtained.

**Figure 10:**
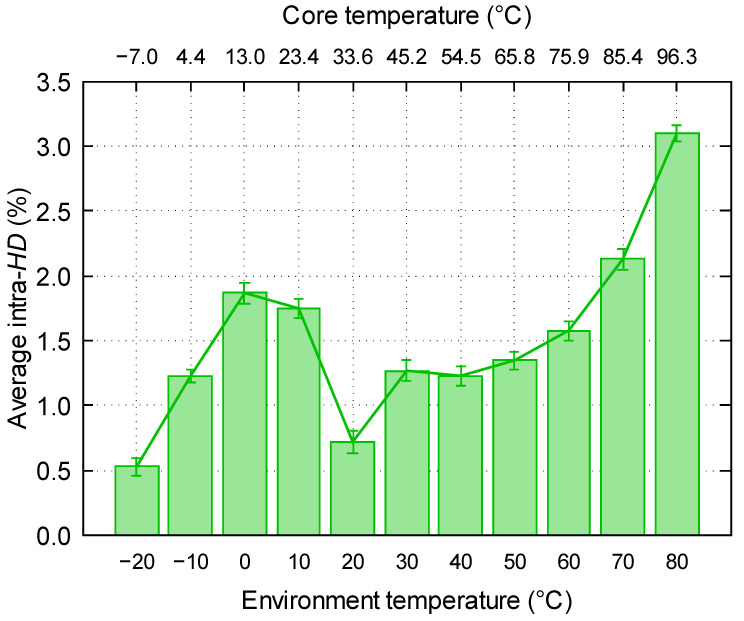
Evolution of intra-*HD* with regard to the “golden key” depending on the temperature for the 200 first same-domain strategy.

As it can be seen in Figure 10, the lowest average hamming distance is obtained for temperatures closer to the temperature of the “golden key”. Furthermore, we have observed that hamming distances further from the hamming distance at room temperature are higher than those closer to the temperature of the “golden key”. In fact, in the range of temperatures studied, the highest hamming distances are obtained for an environment temperature of 70 °C and 80 °C. This experiment shows that the responses do not stray too far from the "golden key" when operating in extreme conditions (−20 °C or 80 °C).

### 5.2. Voltage Variations

Finally, we have studied the effect of changing the supply voltage of the FPGA in the performance of the RO-PUF. For this purpose, the reference voltage of the FPGA ($V_{\mathrm{REF}}$) has been changed in intervals of 10 mV, making the core voltage ($V_{\mathrm{CCINT}}$) vary in intervals of 12.5 mV. To avoid damaging the FPGA, we have varied $V_{\mathrm{CCINT}}$ by a maximum of 10%. Then, we have measured the responses of the RO-PUF at different voltages compared to the response of the PUF at standard voltage conditions, in this case 1.022 V. Again, among the 100 binary sequences obtained from the PUF at 1.022 V, we have obtained the “golden key”.

**Figure 11:**
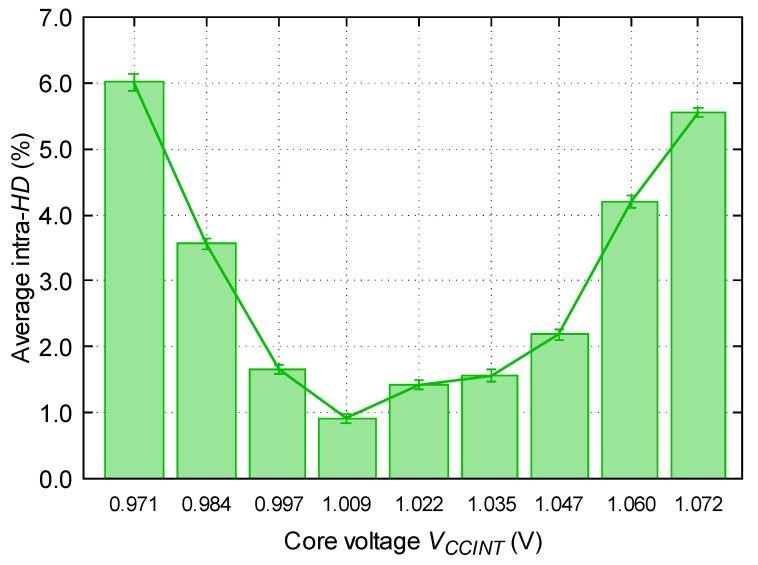
Evolution of intra-*HD* with regard to the “golden key” depending on the core voltage for the 200 first same-domain strategy.

As it is shown in Figure 11, the lowest hamming distance is obtained when the experiment is performed at the voltage of the “golden key”. On the one hand, in the range of voltages from 1.022 to 1.072 V, the hamming distance increases as it increases the voltage. On the other hand, for hamming distances lower than the voltage of the “golden key”, there is no clear trend in the hamming distance as the voltage increases.

However, in general terms, the analysis carried out agrees with the expected, since the more different the operating conditions are with respect to certain conditions, the more bits are different compared to the “golden key”. As it can be seen, when using “extreme” voltage values, the RO-PUF still works fine.

## 6. Conclusions

In this work, a complete analysis is carried out for the optimization of a RO-PUF in an FPGA. As we have shown, the quality of the PUF can be greatly affected depending on some parameters such as the routing of the oscillators, their location, or the type of slice where they are implemented. We have observed that the automatic routing carried out by Vivado is different depending on the position of the LUT in the FPGA.

Firstly, in order to eliminate possible edge effects and other effects due to the existence of physical separations between the different slices of the same FPGA, the routing pattern has been extracted and fixed before the implementation phase. In this way, oscillators implemented in the same type of slice (0 or 1) have analogous routing and therefore, similar frequencies. This study shows the importance of avoiding comparisons of oscillators implemented in different types of slices, since these comparisons decrease the randomness of the response.

Secondly, some oscillator selection strategies are proposed to minimize the remaining effects to improve the performance of a PUF implemented in an FPGA. The key idea is to select oscillators with similar frequencies so that, when comparing oscillators in pairs, the output bit that results from the comparison is not predictable. Among the five strategies analyzed, two strategies stand out as the best strategies due to the high uniqueness, reproducibility, and identifiability obtained: 200 first same-domain, where they have been selected as close oscillators implemented in the same type of slice; and 200 optimal, where those oscillators of the same domain and most similar frequencies have been selected. Both strategies provide great results in terms of identifiability. We conclude that 200 first same-domain is the best strategy to select the oscillators since it does not need a prior analysis of the frequencies of the oscillators. However, the 200 optimal strategy provides a solution for those cases in which it is difficult to fix the routing of the oscillators, just by analyzing a small set of FPGAs.

Regarding the resources and power consumption, all the strategies consume the same power and use the same number of FPGA resources during the authentication process. Although it is true that some strategies such as 200 optimal require a prior study of the frequencies of the oscillators in which it is necessary to implement a high number of oscillators compared to the number of oscillators used to generate the response of the PUF that may increment the resources and power consumption, once the best locations have been determined, it is only necessary to implement the exact oscillators in those specific locations to generate the PUF response. In terms of implementation complexity, the 200 first and 200 random strategies are the easiest to implement in an FPGA since they do not require any prior study of the architecture of the FPGA. On the other hand, the 200 random same-domain and 200 first same-domain strategies are more complicated to implement compared to the previous ones, since it may be necessary to first study how the different types of Slices, LUTs, and CLBs are distributed in a specific FPGA. However, in some areas of the FPGA, some patterns in the location of Slices, LUTs, and CLBs are observed which may facilitate this analysis. Finally, the 200 optimal strategy is the hardest to implement in a real scenario, since it may be necessary to perform a previous analysis of the frequencies of the oscillators in a small set of FPGAs, to determine which are the optimal locations to implement the oscillators. However, this is a study which only needs to be conducted once, so that this analysis could be performed during the enrollment phase of the identification process. The high identifiability obtained shows that the proposed PUF can be used for device authentication purposes, especially to generate a cryptographic key to authenticate IoT devices.

Finally, we have observed that the response of the PUF with the 200 first same-domain strategy is stable facing temperature and voltage variations, highlighting some of the benefits of comparing oscillators in pairs, and making the proposed RO-PUF suitable to be implemented in wireless sensor networks located in harsh environments.

Future lines of research may include to implement other types of PUF architectures or analyze whether, by comparing the frequencies of the oscillators in another way, it is also possible to minimize the effect of the location of the oscillators in the FPGA. This analysis could also be extended to other devices and manufacturers and an analysis of the consumption of the oscillators individually could be carried out to obtain structures of RO-PUFs that also minimize said consumption.

## Figures and Tables

**Table 1:** Average frequency depending on the type of CLB (L or R), type of slice (0 or 1), and type of LUT (M or L) where the oscillator is implemented.

Type of LUT	Frequency (MHz)	
and CLB	Slice(1)	Slice(0)
M_L	*	577.66 ± 0.26
M_R	*	576.97 ± 0.24
L_L	609.81 ± 0.20	580.18 ± 0.28
L_R	605.77 ± 0.22	579.00 ± 0.29

* All Slices(1) are type-L slices.

**Table 2:** Average inter-*HD*, binomial fit parameter (*p*), residual sum of squares of the binomial fit (*RSS*), and Kolmogorov–Smirnov statistic (*D*_K-S_) for each strategy.

Strategy	Average Inter-*HD* (%)	*p*	*RSS*	*D*_K-S_
200 first	2.22 ± 0.10	0.0053	0.0791	1.000000
200 random	18.49 ± 0.16	0.1820	0.0028	0.999515
200 random same-domain	36.08 ± 0.27	0.3616	0.0075	0.838929
200 first same-domain	47.79 ± 0.20	0.4809	0.0014	0.151243
200 optimal	48.42 ± 0.19	0.4856	0.0019	0.114899

**Table 3:** Inter-*HD* for each of the FPGAs and strategies.

Strategy	Intra-*HD* (%)					Average Intra-*HD* (%)
	1	2	3	4	5	
200 first	0.00	0.00	0.00	0.00	0.00	0.00 ± 0.00
200 random	0.11	1.14	0.12	0.41	0.20	0.39 ± 0.17
200 random same-domain	1.11	0.49	0.59	1.26	0.14	0.72 ± 0.18
200 first same-domain	1.29	2.26	1.00	1.29	2.19	0.18 ± 0.23
200 optimal	1.69	2.22	2.08	1.54	2.08	1.92 ± 0.12

**Table 4:** Identifiability metrics: threshold (t_EER_), false rejection rate (*FRR*), false acceptance rate (*FAR*), and equal error rate (*EER*).

Strategy	*t*_*EER*_	*FRR* (*t*_*EER*_)	*FAR*(*t*_*EER*_)	*EER*
200 first	0	0.00	0.59	0.59
200 random	5	3.27 × 10^−6^	9.92 × 10^−5^	9.92 × 10^−5^
200 random same-domain	10	2.03 × 10^−10^	2.35 × 10^−9^	2.35 × 10^−9^
200 first same-domain	16	9.67 × 10^−12^	1.66 × 10^−11^	1.66 × 10^−11^
200 optimal	17	1.10 × 10^−11^	4.28 × 10^−11^	4.28 × 10^−11^

## Data Availability

The data that support the findings of this study are available from the corresponding author upon reasonable request.

## Abbreviations

The following abbreviations are used in this manuscript:

CDF	Cumulative Distribution Function
EER	Equal Error Rate
FAR	False Acceptance Rate
FPGA	Field Programmable Gate Array
FRR	False Rejection Rate
HD	Hamming Distance
LUT	Look-Up Table
MUX	Multiplexer
PUF	Physical Unclonable Function
RO	Ring Oscillator
WSN	Wireless Sensor Network
